# MicroRNA Polymorphisms in Cancer: A Literature Analysis

**DOI:** 10.3390/cancers7030863

**Published:** 2015-09-09

**Authors:** Veronika Pipan, Minja Zorc, Tanja Kunej

**Affiliations:** Department of Animal Science, Biotechnical Faculty, University of Ljubljana, Groblje 3, SI-1230 Domzale, Slovenia; E-Mails: veronika.pipan@gmail.com (V.P); minja.zorc@bf.uni-lj.si (M.Z.)

**Keywords:** microRNA (miRNA), cancer, single nucleotide polymorphisms (SNP)

## Abstract

Single nucleotide polymorphisms (SNPs) located in microRNA (miRNA) genes (miR-SNPs) have attracted increasing attention in recent years due to their involvement in the development of various types of cancer. Therefore, a systematic review on this topic was needed. From 55 scientific publications we collected 20 SNPs, which are located within 18 miRNA encoding genes and have been associated with 16 types of cancer. Among 20 miRNA gene polymorphisms 13 are located within the premature miRNA region, five within mature, and two within mature seed miRNA region. We graphically visualized a network of miRNA-cancer associations which revealed miRNA genes and cancer types with the highest number of connections. Our study showed that, despite a large number of variations currently known to be located within miRNA genes in humans, most of them have not yet been tested for association with cancer. MicroRNA SNPs collected in this study represent only 0.43% of known miRNA gene variations (20/4687). Results of the present study will be useful to researchers investigating the clinical use of miRNAs, such as the roles of miRNAs as diagnostic markers and therapeutic targets.

## 1. Introduction

MicroRNAs (miRNAs) are a class of non-protein coding RNAs that post-transcriptionally regulate expression of the target mRNAs [1]. During miRNA biogenesis, premature miRNA undergoes cleavage in subsequently regulated steps by two enzymes to form mature miRNA. First, pri-miRNA is cleaved into ~70-nt-long pre-miRNA by Drosha and its cofactor DGCR8. Next, Dicer enzyme cleaves pre-miRNA into mature miRNA. The crucial binding location for translational regulation resides in the mature miRNA sequence, more accurately within the nucleotides 2–7 or 2–8 from the 5′ end of the miRNA, called the seed region [2,3]. MicroRNAs are known to be critical post-transcriptional regulators of target mRNA expression in both normal and abnormal biological processes, including cancer. Abnormalities in regulation of miRNAs occur in various types of cancers and are associated with tumor initiation, drug resistance and metastasis [4].

Single nucleotide polymorphisms (SNPs) located in miRNA encoding gene (miR-SNPs) have recently come into focus regarding their possible role in the development of cancer. It is known that miR-SNPs can influence the transcription of the target gene, alter the processing of pri-miRNA or pre-miRNA and affect interaction between miRNA and mRNA [5]. Moreover, miR-SNPs also represent an indispensable pool of novel molecular biomarkers.

Several meta-analyses related with a specific type of cancer or a couple of related types of cancer (for example, various cancers of the digestive tract [6]) have already been performed. Due to recent increasing interest in miR-SNPs, a systematic review on this topic was needed. The goal of the present study was, therefore, to (1) create a list of published miRNA gene SNPs associated with various types of cancer and (2) visualize associations between miRNAs and types of cancer in order to identify miRNA genes and cancer types with a higher number of connections.

## 2. Results and Discussion

**Figure 1:**
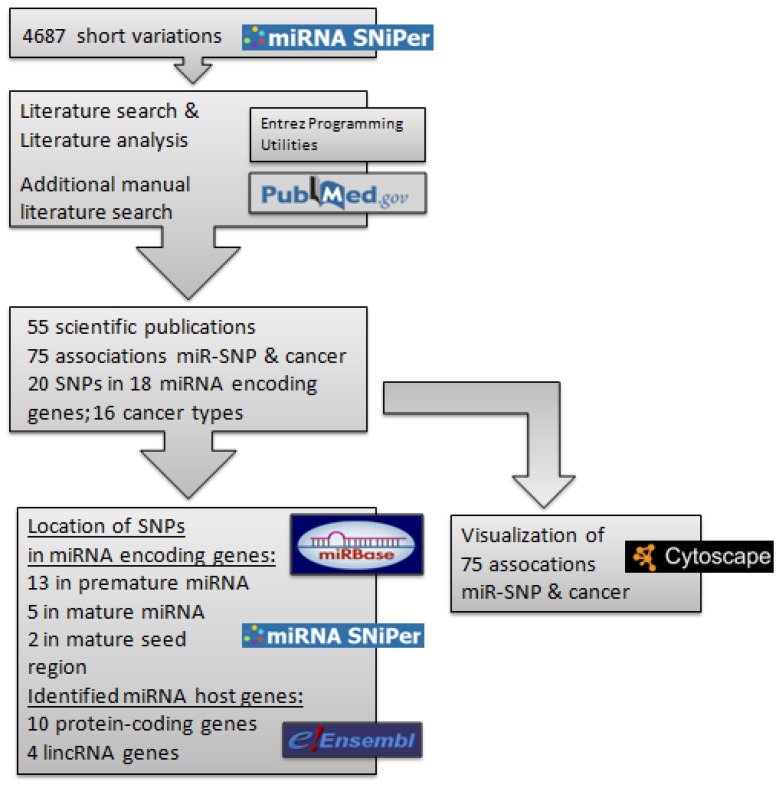
Workflow of the study and main results.

Literature mining was performed to systematically review published miR-SNPs associated with cancer. Seventy-five collected associations between miRNA and types of cancer were then visualized as a network in order to identify hubs with a higher number of connections. Workflow of the study and main results are shown in Figure 1.

### 2.1. List of Cancer Associated Polymorphisms within miRNA Encoding Genes

A publication search was performed in order to create a list of cancer-associated SNPs, which are located within miRNA encoding genes. Among 148 papers collected, 93 were not included in the analysis due to exclusion criteria. The literature analysis of 55 publications, including 16 meta-analyses that involved 70 papers, revealed 75 associations between miR-SNPs and cancers types. The review revealed 20 SNPs within 18 miRNA encoding genes which have been associated with 16 different types of cancer. Details regarding 75 associations are presented in the Supplementary Files (Table S1) including information on the effect of the polymorphism, ethnicity of the study population, study type, number of cases, and controls and reference. Among 20 collected miRNA gene polymorphisms 13 are located within premature miRNA region, five within mature, and two within the mature seed miRNA region (Table 1). All of the tested miRNA polymorphisms are single-nucleotide substitutions, including two multi-allele substitutions (rs12355840, rs895819). Nine polymorphic miRNA genes were tested in single studies, and nine were reported in more than two studies, including *hsa-mir-146a* and *hsa-mir-196a-2*, which were described in 13 and 14 individual studies, respectively.

**Table 1:** Locations of SNPs within miRNA encoding genes and host genes.

miRNA Name	rs Number	Substitution	Location	Genomic Context (Host Genes)	
				Protein-Coding	Non-Coding Transcriptional Unit
hsa-mir-27a	rs11671784	G>A	pre-mature		
	rs895819	T>A, T>C, T>G	pre-mature		
hsa-mir-146a	rs2910164	C>G	seed		CTC-231O11.1 (exon, S)
hsa-mir-149	rs71428439	A>G	pre-mature	*GPC1* (intron, S)	
	rs2292832	T>C	pre-mature	*GPC1* (intron, S)	
hsa-mir-196a-2	rs11614913	C>T	mature	*HOXC6* (intron, S)	
hsa-mir-202	rs12355840	C>A, C>T, C>G	pre-mature		*MIR202HG* (intron/exon, As)
hsa-mir-423	rs6505162	A>C	pre-mature	*NSRP1* (intron, S)	*MIR3184* (intron, S)
hsa-mir-499a	rs3746444	A>G	seed	*MYH7B* (intron, S)	
hsa-mir-603	rs11014002	C>T	pre-mature	*KIAA1217* (intron, S)	
hsa-mir-605	rs2043556	T>C	pre-mature	*PRKG1* (intron, S)	
hsa-mir-608	rs4919510	C>G	mature	*SEMA4G* (intron, S), *MRPL43* (intron, As)	
hsa-mir-612	rs12803915	G>A	pre-mature		*NEAT1* (exon, S)
hsa-mir-618	rs2682818	A>C	pre-mature	*LIN7A* (intron, As)	
hsa-mir-646	rs6513497	T>G	mature		*MIR646HG* (intron, S)
hsa-mir-933	rs79402775	G>A	mature	*ATF2* (intron, As)	
hsa-mir-1206	rs2114358	G>A	pre-mature		*PVT1* (intron, S)
hsa-mir-1307	rs7911488	A>G	pre-mature	*USMG5* (intron, As)	
hsa-mir-3144	rs67106263	G>A	mature		
hsa-mir-5197	rs2042253	T>C	pre-mature		

Host gene names: *GPC1*: Glypican 1, *HOXC6*: Homeobox C6, *MIR202HG*: MIR202 host gene, *NSRP1*: Nuclear speckle splicing regulatory protein 1, MIR3184: microRNA 3184 (*hsa-mir-3184*), MYH7B: Myosin, heavy chain 7B, cardiac muscle, beta, *KIAA1217*, *PRKG1*: Protein kinase, cGMP-dependent, type I, *SEMA4G*: Sema domain, immunoglobulin domain (Ig), transmembrane domain (TM) and short cytoplasmic domain, (semaphorin) 4G, *MRPL43*: Mitochondrial ribosomal protein L43, *NEAT1*: Nuclear paraspeckle assembly transcript 1 (non-protein coding), *LIN7A*: Lin-7 homolog A (*C. elegans*), *MIR646HG*: MIR646 host gene, *ATF2*: Activating transcription factor 2, *PVT1*: Pvt1 oncogene (non-protein coding), *USMG5*: Up-regulated during skeletal muscle growth 5 homolog (mouse). Host gene orientation abbreviation: S: sense, As: antisense.

### 2.2. Visualization of 75 Associations between miR-SNPs and Cancer

**Figure 2:**
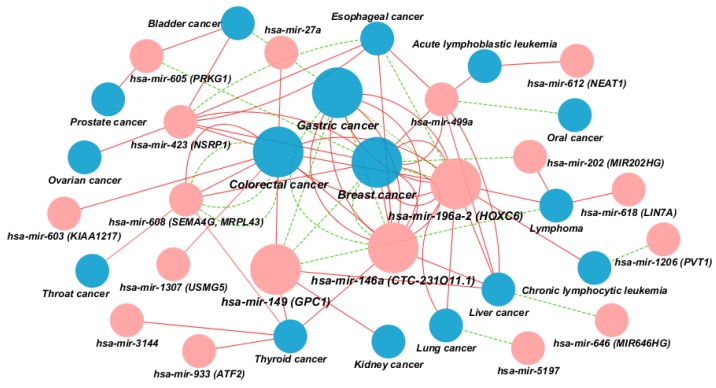
Network representing 75 associatons between 18 miRNA encoding genes and 16 types of cancer. MicroRNA encoding genes are presented as pink nodes while different types of cancer are shown as blue nodes. The size of a node correlates with the number of its associations. Red solid edges represent associations with negative outcome (increased risk of cancer, overall survival risk, unfavorable overall and recurrence-free survival, and increased risk of recurrence and death) and green dashed edges represent associations with positive outcome (decreased risk of cancer, better response to chemotherapy, increased survival, protective against mortality, favorable overall and recurrence-free survival, and decreased risk of recurrence). MicroRNA host genes are presented in parentheses.

A total of 75 associations between miRNA and cancers were visualized as a network as shown in Figure 2. Nodes represent cancer types and miRNA encoding genes according to a list of associations in Table S1. The lines between the nodes depict the effect of the miRNA polymorphism as described in publications.

Among 75 SNP-cancer associations, 50 present increased and 15 decreased risk of a particular cancer. The remaining ten associations include six miR-SNPs associated with a risk role in overall survival in both gastric cancer and non-small cell lung cancer in Asian patients (rs11614913; *hsa-mir-196a-2*) [7], increased risk of recurrence and death in patients with colorectal cancer [8], increased risk of death in Caucasian colorectal cancer patients and decreased risk of death in colorectal cancer patients of African American origin [9], favorable overall and recurrence-free survival in Chinese patients with colorectal cancer [10] and decreased risk of recurrence of colorectal cancer in Caucasians (rs4919510, *hsa-mir-608*) [11], unfavorable overall and recurrence-free survival in Chinese patients with colorectal cancer (rs6505162, *hsa-mir-423*) [10], better response to chemotherapy in Chinese bladder cancer patients (rs11671784, *hsa-mir-27a*) [12], improved overall survival of Chinese patients with T-cell lymphoma (rs2292832, *hsa-mir-149*) [13], and protective role against breast cancer mortality (rs12355840, *hsa-mir-202*) [14]. The visualization revealed miRNAs and types of cancer that have the highest number of connections and, therefore, represent hubs in the larger miRNA-disease network. Among 75 associations, miRNA genes have been most frequently associated with breast cancer (15), colorectal cancer (13), and gastric cancer (11). These three types of cancer are considered to be among the most prevalent nowadays and also among the most studied. MicroRNAs with the highest number of associated cancer types are: *hsa-mir-196a-2* (8), *hsa-mir-149* (7), and *hsa-mir-146a* (6). Identified hubs could be important for further studies investigating the role of miRNA in cancer.

We also gathered information on ethnic origin and the size of tested population (number of cases and controls) (Table S1). More than half of the studies (35 out of 55) were performed on Asian population, including 23 on Chinese populations. Other ethnic origins include Caucasian, African American, Indian, and Jewish Israeli populations. The review also revealed opposing results in different studies for the same SNP. For example, polymorphism rs2910164 is associated with decreased risk of breast cancer in the North Indian population [15], while it is associated with increased risk of breast cancer in the Chinese population [16]. Another example is SNP rs4919510, which has been associated with increased and decreased risk of death in colorectal cancer patients of Caucasian and African American origin, respectively [9]. These examples suggested that the effect of polymorphisms within miRNA genes depends on the ethnicity of the patients. On the other hand, we observed consistency in results of different studies performed in the same ethnic group, especially in Asian and Chinese populations. For example, polymorphism rs2910164 has been associated with increased risk of breast cancer in two independent case-control studies in Chinese populations [16,17]. Similarly, polymorphism rs11614913 has been associated with increased risk of colorectal cancer in two independent meta-analyses in Asian population [18,19]. However, due to a small number of studies it is not possible to draw conclusions about the consistency of the results in other ethnic groups. We also observe great heterogeneity in population size among various meta-analyses and case-control studies which can affect the results of the studies (Table S1). Population size in meta-analyses ranged from 1396 cases/1574 controls [20] to 12,799 cases/14,507 controls [21]. Case-control studies were performed on smaller populations; the size of the tested cases/controls ranged between 102/204 [22] and 1026/1026 [23]. Additional studies of polymorphisms within miRNA encoding genes are needed for a more reliable conclusion about the association of SNPs with cancer risk.

### 2.3. Future Directions

Genome-wide *in silico* screening of the human genome revealed a large number of polymorphisms located within miRNA encoding genes (*n* = 4687). Collected miRNA SNPs in this study (n = 20) represent only 0.43% of all short variations currently known to be located in human miRNome. Most miRNA variations have not yet been tested for associations with phenotypic variability and disease development. MicroRNA polymorphisms could now be tested for association with cancer, particularly those located within both miRNA genes and cancer-associated protein-coding host genes. Among 18 miRNA genes, included in this review, ten genes are located within introns of protein-coding genes. Six miRNA genes overlap non-coding transcriptional units: four miRNA genes overlap regions encoding for ncRNAs (lincRNAs; long intergenic non-coding RNAs), one overlaps another miRNA gene located on the reverse strand, and one overlaps a non-coding host gene located on the reverse strand (Table 1). Protein-coding miRNA host genes, as well as non-coding host genes, could be further explored in search for their potential role in cancer development.

Our literature analysis revealed that research in the field of miRNA polymorphisms currently includes a relatively small number of miR-SNPs and consequently various studies investigate the same miRNA polymorphisms (e.g., rs2910164 and rs11614913). Instead, we suggest that scientists expand the research to a large number of untested miR-SNPs. The majority of published polymorphisms within miRNAs are located either in pre-mature or mature regions, whereas only two are located within the mature seed region. The impact of SNPs, particularly those located within the mature seed region, should also be explored. Collected miRNA SNPs could be now integrated into various bioinformatics tools; for instance, our previously developed bioinformatics tool miRNA SNiPer [24].

## 3. Experimental Section

A list of miRNA SNPs was obtained using miRNA SNiPer tool, version 5 [24,25], which comprises 4687 short variations. The Entrez Programming Utilities (eUtils) was used to retrieve all available articles in NLM’s PubMed databases on miRNA SNPs and cancer. The search terms used in PubMed query were individual miRNA names, reference numbers (rs numbers) of SNPs, and the term “cancer”. We performed additional PubMed searches for scientific papers using search terms “miRNA”, “SNP”, “polymorphism”, and “cancer”. The literature analysis includes scientific papers published until 7/2015. Obtained scientific papers were manually curated in order to determine associations between miRNA SNPs and cancer. Exclusion criteria for collected papers were as follows: (1) no association between miRNA (miR-SNP) and cancer; (2) association with a benign tumor or syndrome with a predisposition for cancer; and (3) individual papers that were already included in meta-analyses, collected in this study. For each study, information was extracted concerning the following characteristics of the studies: the name of miRNA, rs number of SNP, cancer type, effect of polymorphism regarding cancer risk (e.g., increased, decreased risk), ethnicity, type of study (e.g., association study, meta-analysis), number of cases, and controls and reference. A list of SNPs, associated with cancer residing within miRNA genes was created and presented in a table, which can be found in Supplementary Files (Table S1). Cytoscape v3.2.1 [26] was used for graphic visualization of the associations between miRNA genes and cancer types and identification of miRNA genes and cancer types with the highest number of associations. Due to heterogeneity in cancer type nomenclature in papers, the names of cancer types were unified according to the MedlinePlus list of cancers ([27]) before visualization. The miRNA SNiPer tool was used to search location of SNPs within miRNA genes: pre-mature, mature, and seed. Information of miRNA protein-coding host genes and overlapping genomic elements was extracted from Ensembl Genome Browser, version 81 (July 2015). Names of protein-coding and miRNA genes are in accordance with the HGNC gene nomenclature and miRBase, respectively. Ensembl Helpdesk was contacted to clarify the annotation of overlapping genomics elements.

## 4. Conclusions

In conclusion, the present literature analysis provides an overview of miRNA polymorphisms associated with cancer and offers suggestions for further research in this area. Studies of miRNA polymorphisms play an important role in the field of cancer research and will be even more significant in the future. The present list of cancer associated SNPs located in miRNA genes will be useful to researchers investigating clinical use of miRNAs such as the roles of miRNAs as diagnostic markers and therapeutic targets.

## Supplementary Files

Supplementary File 1
