# Supplementary material for: MicroRNA Polymorphisms in Cancer: A Literature Analysis
Source: Cancers (Basel). 2015 Sep 9;7(3):1806–14. doi: 10.3390/cancers7030863 (PMC4586796; doi:10.3390/cancers7030863)
Supplement: Supplementary File 1 [file cancers-07-00863-s001.pdf]

Supplementary Information

Table S1. List of miRNA gene polymorphisms associated with cancer.

miRNA Name	rs Number	Cancer type	Effect	Ethnicity	Study type	No. of cases/controls	Reference
hsa-mir-27a	rs11671784	Bladder cancer	Better response to chemotherapy	Chinese	Expression study (Chemo-sensitivity)	89/n.p.	Deng *et al.*, 2015 [1]
		Gastric cancer	Decreased risk	Han Chinese	Case-control study, meta-analysis	278/278	Song *et al.*, 2014 [2]
	rs895819	Colorectal cancer	Increased risk	Han Chinese	Case-control study	254/238	Cao *et al.*, 2014 [3]
hsa-mir-146a	rs2910164	Breast cancer	Increased risk	Chinese	Case-control study	450/450	He *et al.*, 2015 [4]
		Breast cancer	Increased risk	Caucasian	Meta-analysis, 8 studies	4314/4485	Dai *et al.*, 2015 [5]
		Breast cancer	Decreased risk	North Indian	Case-control study	121 BC, 115 BBD/164	Bansal *et al.*, 2014 [6]
		Breast cancer	Increased risk	Chinese	Case-control study	321/290	Qi *et al.*, 2015 [7]
		Colorectal cancer	Increased risk	Greek	Case-control study	157/299	Dikaiakos *et al.*, 2015 [8]
		Esophageal cancer	Increased risk	Asian	Meta-analysis, 14 studies	6053/6527	Xie *et al.*, 2015 [9]
		Follicular thyroid carcinoma	Increased risk	European	Expression study	39 FTC, 20 FTA/n.p.	Roncati *et al.*, 2014 [10]
		Gastric cancer	Increased risk	Asian	Meta-analysis, 14 studies	6053/6527	Xie *et al.*, 2015 [9]
		Gastric cancer	Decreased risk	Asian, Caucasian	Meta-analysis, 9 studies	4468/6844	Ni *et al.*, 2015 [11]
		Gastric cancer	Decreased risk in subgroups	Asian, Caucasian	Meta-analysis, 13 studies	9044/11762	Xu *et al.*, 2015 [12]
		Gastric cancer	Increased risk	Asian	Meta-analysis, 8 studies	4308/6370	Fu *et al.*, 2014 [13]
		Gastric cancer	Increased risk	Caucasian	Meta-analysis, 7 studies	4112/5811	Xie *et al.*, 2014 [14]
		Gastric cancer	Increased risk	Chinese	Meta-analysis, 9 studies	3885/5396	Xu *et al.*, 2014 [15]
		Hepatocellular carcinoma	Increased risk	Asian	Meta-analysis, 12 studies	4171/4901	Peng *et al.*, 2014 [16]
hsa-mir-149	rs71428439	Clear cell renal cell carcinoma	Increased risk	Han Chinese	Case-control study	1000/1000	Wang *et al.*, 2014 [17]
	rs2292832	Breast cancer	Decreased risk	Chinese	Case-control study	450/450	He *et al.*, 2015 [4]
		Colorectal cancer	Increased risk	Asian	Meta-analysis, 4 studies	1396/1574	Du *et al.*, 2014 [18]
		Gastric cancer	Decreased risk	Asian, Caucasian	Meta-analysis, 13 studies	9044/11762	Xu *et al.*, 2015 [12]
		Hepatocellular carcinoma	Increased risk	Eastern Chinese	Association study	993/992	Wang *et al.*, 2014 [19]
		Papillary thyroid cancer	Increased risk	Han Chinese	Case-control study	838/1006	Wei *et al.*, 2014 [20]
		T-cell lymphoma	Increased survival	Han Chinese	Case-control study	220/n.p.	Li *et al.*, 2014 [21]

Table S1. *Cont.*

miRNA Name	rs Number	Cancer type	Effect	Ethnicity	Study type	No. of cases/controls	Reference
hsa-mir-196a-2	rs11614913	Breast cancer	Decreased risk	Caucasian	Meta-analysis, 10 studies	4618/5590	Dai *et al.*, 2015 [5]
		Breast cancer	Increased risk	North Indian	Case-control study	121 BC, 115 BBD/164	Bansal *et al.*, 2014 [6]
		Breast cancer	Increased risk	Chinese	Case-control study	321/290	Qi *et al.*, 2015 [7]
		Chronic lymphocytic leukemia	Increased risk	West European (Spanish)	Association study	132/391	Martin-Guerrero *et al.*, 2015 [22]
		Colorectal cancer	Increased risk	Asian, Caucasian	Meta-analysis, 15 studies	5648/6607	Xie *et al.*, 2015 [9]
		Colorectal cancer	Increased risk	Asian	Meta-analysis, 9 studies	2209/2803	Wu *et al.*, 2015 [23]
		Colorectal cancer	Increased risk	Asian	Meta-analysis, 6 studies	1754/2430	Xu *et al.*, 2015 [24]
		Esophageal squamous cell carcinoma	Decreased risk	Han Chinese	Case-control study	381/426	Qu *et al.*, 2014 [25]
		Gastric cancer	Decreased risk (CC genotype)	Asian, Caucasian	Meta-analysis, 9 studies	3992/5418	Ni *et al.*, 2015 [11]
		Gastric cancer	Increased risk	Asian, Caucasian	Meta-analysis, 13 studies	9044/11762	Xu *et al.*, 2015 [12]
		Gastric cancer	Overall survival risk	Asian	Meta-analysis, 14 studies	8057/n.p.	Xia *et al.*, 2014 [26]
		Hepatocellular carcinoma	Increased risk	Asian	Case-control study	314/407	Qi *et al.*, 2014 [27]
		Lung cancer	Increased risk	East Asian	Meta-analysis, 7 studies	3705/4099	Fan and Wu, 2014 [28]
		Non-Hodgkin lymphoma	Increased risk	Han Chinese	Case-control study	318/320	Li *et al.*, 2014 [29]
		Non-small cell lung carcinoma	Overall survival risk	Asian	Meta-analysis, 14 studies	8057/n.p.	Xia *et al.*, 2014 [26]
hsa-mir-202	rs12355840	Breast cancer	Protective against mortality	European, Asian, African	Whole-genome sequencing	69/n.p.	Rawlings-Goss *et al.*, 2014 [30]
		Follicular lymphoma	Increased risk	Caucasian, African American, Other	Case-control study	455/527	Hoffman *et al.*, 2013 [31]

Table S1. *Cont.*

miRNA Name	rs Number	Cancer type	Effect	Ethnicity	Study type	No. of cases/controls	Reference
hsa-mir-423	rs6505162	Bladder cancer	Increased risk	Caucasian	Association study	3527/5119	Hu *et al.*, 2014 [32]
		Breast cancer	Increased risk	Chinese	Genetic analysis, preliminary function study	114/114	Zhao *et al.*, 2015 [33]
		Breast cancer	Increased risk	Jewish Israeli	Preliminary study	140 BC, 63 OC/160 (*BRCA1*), 58 BC, 19 OC/48 (*BRCA2*)	Kontorovich *et al.*, 2010 [34]
		Colorectal cancer	Unfavorable overall and recurrence-free survival	Han Chinese	Association Study (Survival analysis)	408/n.p.	Xing *et al.*, 2012 [35]
		Esophageal cancer	Decreased risk	Caucasian	Case-control study	346/346	Ye *et al.*, 2008 [36]
		Esophageal squamous cell carcinoma	Increased risk	Chinese	Case-control study	629/686	Yin *et al.*, 2013 [37]
		Esophageal squamous cell carcinoma	Increased risk	Black, mixed ancestry (South Africa)	Case-control study	565/1000	Wang *et al.*, 2013 [38]
		Ovarian cancer	Increased risk	Jewish Israeli	Preliminary study	140 BC, 63 OC/160 (*BRCA1*), 58 BC, 19 OC/48 (*BRCA2*)	Kontorovich *et al.*, 2010 [34]
hsa-mir-499a	rs3746444	B-cell acute lymphoblastic leukemia	Increased risk	European Caucasian	Association study	213/387	Gutierrez-Camino *et al.*, 2014 [39]
		Breast cancer	Increased risk	Chinese	Case-control study	450/450	He *et al.*, 2015 [4]
		Breast cancer	Increased risk	Caucasian	Meta-analysis, 5 studies	2924/3563	Dai *et al.*, 2015 [5]
		Esophageal cancer	Increased risk	Asian (Iranian and Chinese)	Meta-analysis, 31 studies	12799/14507	Chen *et al.*, 2014 [40]
		Hepatocellular carcinoma	Increased risk	Asian	Case-control study	314/407	Qi *et al.*, 2014 [27]
		Hepatocellular carcinoma	Increased risk	East Chinese	Case-control study	984/991	Ma *et al.*, 2014 [41]
		Oral squamous cell carcinoma	Decreased risk	Chinese	2 case-control studies	155 OSCC, 169 OL, 80 OSF/204; 512 OSCC/668	Hou *et al.*, 2015 [42]
hsa-mir-603	rs11014002	Colorectal cancer	Increased risk	Chinese (Xiaoshan County)	Case-control study	102/204	Wang *et al.*, 2014 [43]

Table S1. *Cont.*

miRNA Name	rs Number	Cancer type	Effect	Ethnicity	Study type	No. of cases/controls	Reference
hsa-mir-605	rs2043556	Bladder cancer	Increased risk	Caucasian	Association study	3527/5119	Hu *et al.*, 2014 [32]
		Breast cancer	Decreased risk	Asian	Meta-analysis, 12 studies	7170/8783	Chen *et al.*, 2014 [44]
		Prostate cancer	Increased risk	320 Asian, 526 Caucasian	Association study	846/n.p.	Huang *et al.*, 2014 [45]
hsa-mir-608	rs4919510	Breast cancer	Increased risk	Chinese	2 case-control studies	1138/1434, 294/500	Huang *et al.*, 2012 [46]
		Colorectal cancer	Favorable overall and recurrence-free survival	Han Chinese	Association study (Survival analysis)	408/n.p.	Xing *et al.*, 2012 [35]
		Colorectal cancer	Decreased risk of recurrence	Caucasian (Czech Republic)	Association study (Survival analysis)	1083/n.p.	Pardini *et al.*, 2015 [47]
		Colorectal cancer	Increased risk of recurrence and death	Caucasian, African American, Other	Association study	1097/n.p.	Lin *et al.*, 2012 [48]
		Colorectal cancer	Increased/ decreased death risk	Caucasian/African-Americans	Case-control study	245/446	Ryan *et al.*, 2012 [49]
		Nasopharyngeal carcinoma	Increased risk	Chinese	Case-control study	906/1072	Qiu *et al.*, 2015 [50]
		Papillary thyroid cancer	Increased risk	Han Chinese	Association study	828 PTC, 488 BN/1038	Wei *et al.*, 2014 [51]
hsa-mir-612	rs12803915	B-cell acute lymphoblastic leukemia	Increased risk	European Caucasian	Association study	213/387	Gutierrez-Camino *et al.*, 2014 [39]
hsa-mir-618	rs2682818	Follicular lymphoma	Increased risk	Caucasian, African-American, other	Case-control study	455/527	Fu *et al.*, 2014 [52]
hsa-mir-646	rs6513497	Hepatocellular carcinoma	Decreased risk	Chinese	Case-control study	997/993	Wang *et al.*, 2014 [53]
hsa-mir-933	rs79402775	Papillary thyroid cancer	Increased risk	Han Chinese	Association study	828 PTC, 488 BN/1038	Wei *et al.*, 2014 [51]
hsa-mir-1206	rs2114358	Chronic lymphocytic leukemia	Decreased risk	West European (Spanish)	Association study	132/391	Martin-Guerrero *et al.*, 2015 [22]
hsa-mir-1307	rs7911488	Colorectal cancer	Increased risk	Chinese (Jiangsu province)	Case-control study	1026/1026	Tang *et al.*, 2015 [54]
hsa-mir-3144	rs67106263	Papillary thyroid cancer	Increased risk	Han Chinese	Association study	828 PTC, 488 BN/1038	Wei *et al.*, 2014 [51]
hsa-mir-5197	rs2042253	Non-small cell lung cancer	Decreased risk	Caucasian	Association study	452 early-, 526 late-stage cases/0	Zhao *et al.*, 2014 [55]

BC—Breast cancer, BBD—Benign breast disease, FTC—Follicular thyroid cancer, FTA—Follicular thyroid adenoma, OL—Oral leukoplakia, OSF—Oral submucous fibrosis, PTC—Papillary thyroid cancer, OC—Ovarian cancer, BN—Benign thyroid tumor, n.p.—data not provided.

References

1. Deng, Y.; Bai, H.; Hu, H. Rs11671784 g/a variation in mir-27a decreases chemo-sensitivity of bladder cancer by decreasing mir-27a and increasing the target runx-1 expression. *Biochem. Biophys. Res. Commun.* **2015**, *458*, 321–327.
2. Song, B.; Yan, G.; Hao, H.; Yang, B. Rs11671784 G/A and rs895819 A/G polymorphisms inversely affect gastric cancer susceptibility and miR-27a expression in a Chinese population. *Med. Sci. Monit.* **2014**, *20*, 2318–2326.
3. Cao, Y.; Hu, J.; Fang, Y.; Chen, Q.; Li, H. Association between a functional variant in microRNA-27a and susceptibility to colorectal cancer in a Chinese Han population. *Genet. Mol. Res.* **2014**, *13*, 7420–7427.
4. He, B.; Pan, Y.; Xu, Y.; Deng, Q.; Sun, H.; Gao, T.; Wang, S. Associations of polymorphisms in microRNAs with female breast cancer risk in chinese population. *Tumour Biol.* **2015**, *36*, 4575–4582.
5. Dai, Z.J.; Shao, Y.P.; Wang, X.J.; Xu, D.; Kang, H.F.; Ren, H.T.; Min, W.L.; Lin, S.; Wang, M.; Song, Z.J. Five common functional polymorphisms in microRNAs (rs2910164, rs2292832, rs11614913, rs3746444, rs895819) and the susceptibility to breast cancer: Evidence from 8361 cancer cases and 8504 controls. *Curr. Pharm. Des.* **2015**, *21*, 1455–1463.
6. Bansal, C.; Sharma, K.L.; Misra, S.; Srivastava, A.N.; Mittal, B.; Singh, U.S. Common genetic variants in pre-microRNAs and risk of breast cancer in the north indian population. *Ecancermedicalscience* **2014**, *8*, 473.
7. Qi, P.; Wang, L.; Zhou, B.; Yao, W.J.; Xu, S.; Zhou, Y.; Xie, Z.B. Associations of miRNA polymorphisms and expression levels with breast cancer risk in the chinese population. *Genet. Mol. Res.* **2015**, *14*, 6289–6296.
8. Dikaiakos, P.; Gazouli, M.; Rizos, S.; Zografos, G.; Theodoropoulos, G.E. Evaluation of genetic variants in miRNAs in patients with colorectal cancer. *Cancer Biomark.* **2015**, *15*, 163–168.
9. Xie, M.; Li, Y.; Wu, J. A risk of digestive tract neoplasms susceptibility in miR-146a and mir-196a2. *Fam. Cancer* **2015**, *14*, 229–239.
10. Roncati, L.; Pignatti, E.; Vighi, E.; Magnani, E.; Kara, E.; Rochira, V.; Carani, C.; Simoni, M.; Maiorana, A. Pre-miR146a expression in follicular carcinomas of the thyroid. *Pathologica* **2014**, *106*, 58–60.
11. Ni, Q.; Ji, A.; Yin, J.; Wang, X.; Liu, X. Effects of two common polymorphisms rs2910164 in miR-146a and rs11614913 in miR-196a2 on gastric cancer susceptibility. *Gastroenterol. Res. Pract.* **2015**, *2015*, 764163.
12. Xu, Q.; Liu, J.W.; Yuan, Y. Comprehensive assessment of the association between miRNA polymorphisms and gastric cancer risk. *Mutat. Res. Rev. Mutat. Res.* **2015**, *763*, 148–160.
13. Fu, B.; Song, P.; Lu, M.; Wang, B.; Zhao, Q. The association between miR-146a gene rs2910164 polymorphism and gastric cancer risk: A meta-analysis. *Biomed. Pharmacother.* **2014**, *68*, 923–928.
14. Xie, W.Q.; Tan, S.Y.; Wang, X.F. MiR-146a rs2910164 polymorphism increases risk of gastric cancer: A meta-analysis. *World J. Gastroenterol.* **2014**, *20*, 15440–15447.
15. Xu, Z.; Zhang, L.; Cao, H.; Bai, B. MiR-146a rs2910164 G/C polymorphism and gastric cancer susceptibility: A meta-analysis. *BMC Med. Genet.* **2014**, *15*, 117.

16. Peng, Q.; Li, S.; Lao, X.; Chen, Z.; Li, R.; Deng, Y.; Qin, X. The association of common functional polymorphisms in miR-146a and miR-196a2 and hepatocellular carcinoma risk: Evidence from a meta-analysis. *Medicine (Baltimore)* **2014**, *93*, e252.
17. Wang, Z.; Wei, M.; Ren, Y.; Liu, H.; Wang, M.; Shi, K.; Jiang, H. MiR149 rs71428439 polymorphism and risk of clear cell renal cell carcinoma: A case-control study. *Tumour Biol.* **2014**, *35*, 12127–12130.
18. Du, W.; Ma, X.L.; Zhao, C.; Liu, T.; Du, Y.L.; Kong, W.Q.; Wei, B.L.; Yu, J.Y.; Li, Y.Y.; Huang, J.W.; *et al.* Associations of single nucleotide polymorphisms in miR-146a, miR-196a, miR-149 and miR-499 with colorectal cancer susceptibility. *Asian Pac. J. Cancer Prev.* **2014**, *15*, 1047–1055.
19. Wang, R.; Zhang, J.; Ma, Y.; Chen, L.; Guo, S.; Zhang, X.; Wu, L.; Pei, X.; Liu, S.; Wang, J.; *et al.* Association study of miR-149 rs2292832 and miR-608 rs4919510 and the risk of hepatocellular carcinoma in a large-scale population. *Mol. Med. Rep.* **2014**, *10*, 2736–2744.
20. Wei, W.J.; Lu, Z.W.; Li, D.S.; Wang, Y.; Zhu, Y.X.; Wang, Z.Y.; Wu, Y.; Wang, Y.L.; Ji, Q.H. Association of the miR-149 rs2292832 polymorphism with papillary thyroid cancer risk and clinicopathologic characteristics in a chinese population. *Int. J. Mol. Sci.* **2014**, *15*, 20968–20981.
21. Li, X.; Tian, X.; Zhang, B.; Chen, J. Polymorphisms in microRNA-related genes are associated with survival of patients with T-cell lymphoma. *Oncologist* **2014**, *19*, 243–249.
22. Martin-Guerrero, I.; Gutierrez-Camino, A.; Lopez-Lopez, E.; Bilbao-Aldaiturriaga, N.; Pombar-Gomez, M.; Ardanaz, M.; Garcia-Orad, A. Genetic variants in mirna processing genes and pre-miRNAs are associated with the risk of chronic lymphocytic leukemia. *PLoS ONE* **2015**, *10*, e0118905.
23. Wu, Y.; Hao, X.; Feng, Z.; Liu, Y. Genetic polymorphisms in mirnas and susceptibility to colorectal cancer. *Cell Biochem. Biophys.* **2015**, *71*, 271–278.
24. Xu, L.; Tang, W. Associations of polymorphisms in miR-196a2, miR-146a and miR-149 with colorectal cancer risk: A meta-analysis. *Pathol. Oncol. Res.* **2015**, in press.
25. Qu, Y.; Qu, H.; Luo, M.; Wang, P.; Song, C.; Wang, K.; Zhang, J.; Dai, L. MicroRNAs related polymorphisms and genetic susceptibility to esophageal squamous cell carcinoma. *Mol. Genet. Genomics* **2014**, *289*, 1123–1130.
26. Xia, L.; Ren, Y.; Fang, X.; Yin, Z.; Li, X.; Wu, W.; Guan, P.; Zhou, B. Prognostic role of common microrna polymorphisms in cancers: Evidence from a meta-analysis. *PLoS ONE* **2014**, *9*, e106799.
27. Qi, J.H.; Wang, J.; Chen, J.; Shen, F.; Huang, J.T.; Sen, S.; Zhou, X.; Liu, S.M. High-resolution melting analysis reveals genetic polymorphisms in microRNAs confer hepatocellular carcinoma risk in chinese patients. *BMC Cancer* **2014**, *14*, 643.
28. Fan, X.; Wu, Z. Effects of four single nucleotide polymorphisms in microrna-coding genes on lung cancer risk. *Tumour Biol.* **2014**, *35*, 10815–10824.
29. Li, T.; Niu, L.; Wu, L.; Gao, X.; Li, M.; Liu, W.; Yang, L.; Liu, D. A functional polymorphism in microRNA-196a2 is associated with increased susceptibility to non-hodgkin lymphoma. *Tumour Biol.* **2015**, *36*, 3279–3284.
30. Rawlings-Goss, R.A.; Campbell, M.C.; Tishkoff, S.A. Global population-specific variation in mirna associated with cancer risk and clinical biomarkers. *BMC Med. Genomics* **2014**, *7*, 53.

31. Hoffman, A.E.; Liu, R.; Fu, A.; Zheng, T.; Slack, F.; Zhu, Y. Targetome profiling, pathway analysis and genetic association study implicate miR-202 in lymphomagenesis. *Cancer Epidemiol. Biomark. Prev.* **2013**, *22*, 327–336.
32. Hu, Y.; Yu, C.Y.; Wang, J.L.; Guan, J.; Chen, H.Y.; Fang, J.Y. MicroRNA sequence polymorphisms and the risk of different types of cancer. *Sci. Rep.* **2014**, *4*, 3648.
33. Zhao, H.; Gao, A.; Zhang, Z.; Tian, R.; Luo, A.; Li, M.; Zhao, D.; Fu, L.; Dong, J.T.; Zhu, Z. Genetic analysis and preliminary function study of miR-423 in breast cancer. *Tumour Biol.* **2015**, *36*, 4763–4771.
34. Kontorovich, T.; Levy, A.; Korostishevsky, M.; Nir, U.; Friedman, E. Single nucleotide polymorphisms in mirna binding sites and miRNA genes as breast/ovarian cancer risk modifiers in jewish high-risk women. *Int. J. Cancer* **2010**, *127*, 589–597.
35. Xing, J.; Wan, S.; Zhou, F.; Qu, F.; Li, B.; Myers, R.E.; Fu, X.; Palazzo, J.P.; He, X.; Chen, Z.; *et al.* Genetic polymorphisms in pre-microRNA genes as prognostic markers of colorectal cancer. *Cancer Epidemiol. Biomark. Prev.* **2012**, *21*, 217–227.
36. Ye, Y.; Wang, K.K.; Gu, J.; Yang, H.; Lin, J.; Ajani, J.A.; Wu, X. Genetic variations in microRNA-related genes are novel susceptibility loci for esophageal cancer risk. *Cancer Prev. Res. (Phila)* **2008**, *1*, 460–469.
37. Yin, J.; Wang, X.; Zheng, L.; Shi, Y.; Wang, L.; Shao, A.; Tang, W.; Ding, G.; Liu, C.; Liu, R.; *et al.* Hsa-miR-34b/c rs4938723 T>C and hsa-miR-423 rs6505162 C>A polymorphisms are associated with the risk of esophageal cancer in a chinese population. *PLoS ONE* **2013**, *8*, e80570.
38. Wang, Y.; Vogelsang, M.; Schäfer, G.; Matejcic, M.; Parker, M.I. MicroRNA polymorphisms and environmental smoke exposure as risk factors for oesophageal squamous cell carcinoma. *PLoS ONE* **2013**, *8*, e78520.
39. Gutierrez-Camino, A.; Lopez-Lopez, E.; Martin-Guerrero, I.; Piñan, M.A.; Garcia-Miguel, P.; Sanchez-Toledo, J.; Carbone Bañeres, A.; Uriz, J.; Navajas, A.; Garcia-Orad, A. Noncoding RNA-related polymorphisms in pediatric acute lymphoblastic leukemia susceptibility. *Pediatr. Res.* **2014**, *75*, 767–773.
40. Chen, C.; Yang, S.; Chaugai, S.; Wang, Y.; Wang, D.W. Meta-analysis of hsa-miR-499 polymorphism (rs3746444) for cancer risk: Evidence from 31 case-control studies. *BMC Med. Genet.* **2014**, *15*, 126.
41. Ma, Y.; Wang, R.; Zhang, J.; Li, W.; Gao, C.; Liu, J.; Wang, J. Identification of miR-423 and miR-499 polymorphisms on affecting the risk of hepatocellular carcinoma in a large-scale population. *Genet. Test. Mol. Biomark.* **2014**, *18*, 516–524.
42. Hou, Y.Y.; Lee, J.H.; Chen, H.C.; Yang, C.M.; Huang, S.J.; Liou, H.H.; Chi, C.C.; Tsai, K.W.; Ger, L.P. The association between miR-499a polymorphism and oral squamous cell carcinoma progression. *Oral Dis.* **2015**, *21*, 195–206.
43. Wang, F.J.; Ding, Y.; Mao, Y.Y.; Jing, F.Y.; Zhang, Z.Y.; Jiang, L.F.; Guo, J.F.; Sun, X.J.; Jin, M.J.; Chen, K. Associations between hsa-miR-603 polymorphism, lifestyle-related factors and colorectal cancer risk. *Cancer Biomark.* **2014**, *14*, 225–231.
44. Chen, Q.H.; Wang, Q.B.; Zhang, B. Ethnicity modifies the association between functional microRNA polymorphisms and breast cancer risk: A huge meta-analysis. *Tumour Biol.* **2014**, *35*, 529–543.

45. Huang, S.P.; Lévesque, E.; Guillemette, C.; Yu, C.C.; Huang, C.Y.; Lin, V.C.; Chung, I.C.; Chen, L.C.; Laverdière, I.; Lacombe, L.; *et al.* Genetic variants in microRNAs and microRNA target sites predict biochemical recurrence after radical prostatectomy in localized prostate cancer. *Int. J. Cancer* **2014**, *135*, 2661–2667.
46. Huang, A.J.; Yu, K.D.; Li, J.; Fan, L.; Shao, Z.M. Polymorphism rs4919510:C>G in mature sequence of human microRNA-608 contributes to the risk of her2-positive breast cancer but not other subtypes. *PLoS ONE* **2012**, *7*, e35252.
47. Pardini, B.; Rosa, F.; Naccarati, A.; Vymetalkova, V.; Ye, Y.; Wu, X.; di Gaetano, C.; Buchler, T.; Novotny, J.; Matullo, G.; *et al.* Polymorphisms in microRNA genes as predictors of clinical outcomes in colorectal cancer patients. *Carcinogenesis* **2015**, *36*, 82–86.
48. Lin, M.; Gu, J.; Eng, C.; Ellis, L.M.; Hildebrandt, M.A.; Lin, J.; Huang, M.; Calin, G.A.; Wang, D.; Dubois, R.N.; *et al.* Genetic polymorphisms in microRNA-related genes as predictors of clinical outcomes in colorectal adenocarcinoma patients. *Clin. Cancer Res.* **2012**, *18*, 3982–3991.
49. Ryan, B.M.; McClary, A.C.; Valeri, N.; Robinson, D.; Paone, A.; Bowman, E.D.; Robles, A.I.; Croce, C.; Harris, C.C. Rs4919510 in hsa-miR-608 is associated with outcome but not risk of colorectal cancer. *PLoS ONE* **2012**, *7*, e36306.
50. Qiu, F.; Yang, L.; Zhang, L.; Yang, X.; Yang, R.; Fang, W.; Wu, D.; Chen, J.; Xie, C.; Huang, D.; *et al.* Polymorphism in mature microRNA-608 sequence is associated with an increased risk of nasopharyngeal carcinoma. *Gene* **2015**, *565*, 180–186.
51. Wei, W.J.; Wang, Y.L.; Li, D.S.; Wang, Y.; Wang, X.F.; Zhu, Y.X.; Pan, X.D.; Wang, Z.Y.; Wu, Y.; Jin, L.; *et al.* Association study of single nucleotide polymorphisms in mature microRNAs and the risk of thyroid tumor in a Chinese population. *Endocrine* **2014**, doi:10.1007/s12020-014-0467-8.
52. Fu, A.; Hoffman, A.E.; Liu, R.; Jacobs, D.I.; Zheng, T.; Zhu, Y. Targetome profiling and functional genetics implicate miR-618 in lymphomagenesis. *Epigenetics* **2014**, *9*, 730–737.
53. Wang, R.; Zhang, J.; Jiang, W.; Ma, Y.; Li, W.; Jin, B.; Hu, H.; Wang, J.; Liu, Y.; Liu, J. Association between a variant in microRNA-646 and the susceptibility to hepatocellular carcinoma in a large-scale population. *Sci. World J.* **2014**, *2014*, 312704.
54. Tang, R.; Qi, Q.; Wu, R.; Zhou, X.; Wu, D.; Zhou, H.; Mao, Y.; Li, R.; Liu, C.; Wang, L.; *et al.* The polymorphic terminal-loop of pre-miR-1307 binding with MBNL1 contributes to colorectal carcinogenesis via interference with Dicer1 recruitment. *Carcinogenesis* **2015**, *36*, 867–875.
55. Zhao, Y.; Wei, Q.; Hu, L.; Chen, F.; Hu, Z.; Heist, R.S.; Su, L.; Amos, C.I.; Shen, H.; Christiani, D.C. Polymorphisms in microRNAs are associated with survival in non-small cell lung cancer. *Cancer Epidemiol. Biomark. Prev.* **2014**, *23*, 2503–2511.

© 2015 by the authors; licensee MDPI, Basel, Switzerland. This article is an open access article distributed under the terms and conditions of the Creative Commons Attribution license (http://creativecommons.org/licenses/by/4.0/).
